# The Control of Hospital Expenditure: The Budget or Estimate System

**Published:** 1922-11

**Authors:** Joseph E. Stone

**Affiliations:** (Accountant, St. Thomas's Hospital, London)


### THE CONTROL OF HOSPITAL EXPENDITURE: THE BUDGET OR ESTIMATE SYSTEM.

By JOSEPH E. STONE, F.S.A.A., F.S.S., F.R.Econ.S. (Accountant, St. Thomas's Hospital, London).

THE Voluntary Hospitals Commission in their pamphlet "Notes on Hospital Management" (V.H. Com. 6) state that a system of rationing departments is valuable as a moral factor in keeping down expenditure, and that every hospital should prepare an annual budget. The subject of budgets is one of considerable importance to hospitals, especially so at the present time when strenuous efforts are being made to keep expenditure down as low as possible without in any way impairing efficiency. Budgets in conjunction with properly prepared comparative statements form one of the most efficient means of control over expenditure. Further, a well-thought-out system enables the finance committee to look ahead intelligently, as it is distinguished from pure guesswork in that it rests upon extensive analysis and past records for guidance.

Budgets are really "Accounts in Advance." They involve three important steps: First, there is the collection of data; then comes the analysis of the past records to determine their inter-relation, which work is made easier by the use of graphic charts; and finally there is the application of the fundamental principles discovered by analysis to determine the future financial position of the Hospital. The underlying principle is to ascertain what amount, in addition to receipts from known and certain sources, will be sufficient to meet the expenditure estimated to be incurred during a certain future period, which may or may not be a year, and so to frame the details that comparison will be facilitated between the proposed transactions as set out and the actual transactions. Keeping in view the main purpose of budgets—viz., the control of expenditure—it is desirable that full estimates should be prepared. In some cases, particularly the less important services, the estimates may show only the aggregate sum required. This is scarcely a matter which requires or admits of definite regulations being laid down here, but it is certainly essential that all the principal classes of expenditure of which the aggregate in each case is composed should be set out separately, in order that any tendency to increased expenditure or extravagance may be readily located. The aim should be to give each committee of the hospital such estimates as will, whilst not being clouded with detail, show clearly the various items of expenditure grouped under appropriate headings, which for convenience and purposes of subsequent comparison, are generally the headings of the various nominal accounts in the expenditure ledger which go to make up the expenditure side of the final income and expenditure account of the revised uniform system.

To conform with the Revised Uniform System, hospital accounts are in certain cases required to be kept upon what is known as the income and expenditure system, as distinct from that known as receipts and payments, and it is therefore essential that the budget should be considered from this point of view. The receipts and payments system, although favoured by many hospital accountants by reason of its simplicity, is generally far from satisfactory. It has the tendency to lessen the control of the finance committee over the spending operations of the various committees, as it is quite easy for a committee finding itself coming to the end of its resources to defer the passing, and therefore payment, of its accounts until the next financial period, when its estimates may be unduly inflated owing to the inclusion of items which correctly belonged to the period just completed.

A statement should be prepared for each committee, and, after consideration and approval by each, these statements should be incorporated into one grand summary budget, and presented to the finance committee for their consideration and approval. This summary may take the following form:—

GRAND SUMMAR BUDGET. Year ending............192....

| Heading of account. | Estimate for current year. | Actual expenditure (or income) to end of November. | Estimated further expenditure (or income) for December. | Accruing liabilities (or assets) at Dec. 31. | Total expenditure (or income) for year. | Comparison. | | Estimate for next year. | Increase. | Decrease |
|---|---|---|---|---|---|---|---|---|---|---|
| | | | | | | Saving. | Overspent. | | On actual expenditure (or income). | |
| | £ | £ | £ | £ | £ | £ | £ | £ | £ | £ |

When finally approved by this committee, the amounts are entered at the head of each column of the respective accounts in the expenditure ledger, so as to render frequent comparison a matter only of seconds. The various committees are also informed of the amounts of expenditure sanctioned for their respective departments. Periodical returns comparing the expenditure with the budget should be made by the accountant to the finance committee at least quarterly, in order that control may be maintained over all expenditure *throughout the year*. The return should aim at bringing out the *true position for the period that has elapsed*. This, as the following form will readily show, can be done only by the expenditure of considerable time and care. The form recommended is as follows:—

Statement Showing Actual Expenditure Compared with Estimated Expenditure. ..........months to..........192....

| Heading of account. | Estimate for year. | Proportion of estimate to end of present month. | Actual expenditure to end of present month. | Comparison of expenditure with proportion of estimate. | | Balance of year's estimate remaining unspent. | Remarks. |
|---|---|---|---|---|---|---|---|
| | | | | Saving. | Overspent. | | |
| | £ | £ | £ | £ | £ | £ | |

It will be seen from the above statements that all information necessary for efficient control is provided, and, if the procedure is systematically carried out and energetically supervised by the accountant and the finance committee, it represents the best known method of obtaining a *real* control on over-expenditure and extravagance.

Looking at the subject of budgets from the income point of view, we find another reason for conducting hospital finance on such a system or basis, in that it provides to a great extent a control over the amounts receivable from patients. When the certain income is ascertained—such as grants from central funds, interest or dividends on investments, rents from properties, payments by Approved Societies and Local Authorities, subscriptions, &c.; by deducting the total of these items from the total expenditure estimated to be incurred we arrive at a balance of expenditure. This represents the amount which must be recovered from patients, their relatives, or other people or companies interested in the patients, if the income is to equal the expenditure, and so balance the account. When this balance figure is known, it should be spread over the number of available beds, and a very simple calculation then gives the amount required to be raised per bed. *This is the average amount per patient which must be aimed at.* A graph should be constructed showing, weekly or monthly, the amount aimed at and the actual amount collected, and a constant review of this graph should be made to see that the average is being kept up. The staff responsible for obtaining payments from patients should be shown what is required of them, and they should be kept constantly informed of the progress made in the collection and by how much the collection falls short of the average amount required. Haphazard methods are not conducive to efficiency in the collection of money from patients; the staff should be given something to aim at, and it should be remembered that the higher they aim the higher the results they are likely to gain.

A word on hospital accounting generally may not be thought out of keeping when considering the subject of budgets. For the latter to be prepared on systematic lines it is a matter of prime importance that the books of account should be complete and fully adapted to their purpose. It is doubtful if the varying schemes of accounting at present in use are always supported by records of a sufficiently comprehensive kind, or that all hospitals recognise the necessity of keeping their accounts in such a manner that they can be readily set out in a clear and coherent statement, susceptible of check in detail through connected and plain channels from the earliest to the latest transaction. In particular it may be mentioned that in some cases there seems to be a failure to appreciate the importance of maintaining records of the many items of income on the one side, and, on the other, of linking up and bringing into direct relation such transactions as involve the acquisition of stores by the disbursement of cash.

This question of budgets and books of account does not admit of treatment in detail here, but it is well to impress in general terms a sense of the importance of these matters on all who have to deal with hospital accounts and the control of expenditure.

